# Gendered Authorship and Demographic Research: An Analysis of 50 Years of *Demography*

**DOI:** 10.1007/s13524-016-0482-x

**Published:** 2016-07-11

**Authors:** Sandra Krapf, Michaela Kreyenfeld, Katharina Wolf

**Affiliations:** 1 University of Cologne, Cologne, Germany; 2 Max Planck Institute for Demographic Research, Rostock, Germany; 3 Hertie School of Governance, Berlin, Germany; 4 Population Research Centre, Faculty of Spatial Sciences, University of Groningen, Groningen, The Netherlands

**Keywords:** Demography, Publication practices, Female authorship, Gender, Bibliometrics

## Abstract

*Demography*, the official journal of the Population Association of America, has been given the highest rating among demographic journals by the Social Sciences Citation Index (SSCI). Our aim here is to investigate the development of research subfields and female authorship in *Demography* over the last 50 years. We find that female authorship in *Demography* has risen considerably since the 1980s and that currently a woman is about as likely as a man to be the sole or the first author of a paper published in the journal. However, we find some differences by subfield. Women seem to be overrepresented in the “family and household” research subfield but underrepresented in the “mortality and health” and “data and methods” categories.

## Introduction

Considerable evidence suggests that women are now outperforming men in formal education in most industrialized countries (OECD 2012). In the United States, more women than men are earning Master’s and PhD degrees (Gonzales et al. 2013). Tower et al. (2007), who examined the publication output of women and men in top journals listed in the Thompson ISI index, even concluded that male and female researchers have reached parity in publication output, when the percentage of women in the respective academic field is controlled for. However, more recent studies on women’s academic performance have challenged the idea that the gender gap in publication output has narrowed (Abramo et al. 2009; West et al. 2013). These critical voices have called for a more nuanced exploration of gender and scientific authorship. West et al. (2013) reported that men often hold the prestigious position of first author and that even in more gender-equal fields, gendered authorship is still prevalent in certain subfields. Because the number and the quality of scientific publications are key elements of career advancement in science (Ramsden 1994), it is important that we understand the gendered patterns in research productivity.

At first glance, the gender balance in demographic research seems to be better than it is in other fields of research: 41.9 % of all authors of demographic studies published from 1990 to 2011 are women (West et al. 2013:2).^1^ However, we have no information on the question of whether the increase in female authorship was accompanied by an equal increase in the share of female first or sole authors in demographic research. We also do not know whether there is gender segregation by demographic subfields. We seek to contribute to this research by exploring the development over time of gender gaps in scholarly authorship within subfields of demography. Specifically, we focus on all issues of *Demography*, the flagship journal of the Population Association of America (PAA), which was launched in 1964 and recently celebrated its 50th anniversary. Although previous studies have examined the patterns of publication activities in *Demography* (Teachman et al. 1993; Watkins 1993), no study has explored recent trends. Here, we examine the question of whether there is a gendered pattern of publication by demographic subfield, such as mortality, fertility, or family and household research. We also investigate whether men and women are equally likely to hold the prestigious position of first author for papers with multiple authors. Our empirical analyses are based on the bibliometric information on each article published in *Demography* for the period 1964 to 2014. However, we note two main caveats of our analysis. First, the analysis of articles of a single journal does not allow us to draw conclusions for the entire field of demography. An analysis of all demographic journals, and particularly of those published in other countries, may lead to different outcomes. Second, we assigned articles manually to different subcategories. We believe that our procedure is superior to a computerized classification procedure for reasons discussed in more detail later in the article. However, in several cases, we could not assign a paper unambiguously to a single research subfield.

## Background

The female-male ratio in publications has frequently been used as a bellwether for gender equality and women’s advancement in science. Although the research results are somewhat mixed, the share of authors who are women appears to have increased considerably in recent decades in several fields and across countries (Østby et al. (2013) for the field of international relations; Mauleon et al. (2013) for Spain; Woerdeman and Rodgers (2006) for the Netherlands and the United Kingdom). However, some studies (Abramo et al. 2009; Leahey 2006; West et al. 2013) have challenged the view that the gender gap in scientific publication has disappeared. These authors have argued that most of the studies that found no disparities examined large research fields while systematically overlooking gender imbalances in more narrowly defined subfields. For example, in sociological publications, female authors are overrepresented in subfields such as gender and early childhood but are underrepresented in other subfields (West et al. 2013:3). In an investigation of gender differences in subfields of economics research, Dolado et al. (2012) found that although 37 % of the researchers who earned a PhD between 1996 and 2005 in the top 50 departments in the fields of health, education, and welfare are women, less than 20 % of faculty in the subfield of mathematical and quantitative methods are women. Authorship practices also appear to differ by gender. West et al. (2013) reported, for example, that men predominate in lead authorship.

Several mechanisms that might result in a gender imbalance in research subfields have been identified (for a discussion, see Dolado et al. 2012). The arguments regarding these mechanisms are very much in line with those made in research on occupational sex segregation (Blau et al. 2013; Reskin 1993; Trappe and Rosenfeld 2004). For our research question, two arguments seem to be of specific relevance. First, women may self-select into particular areas of research—such as subfields of early education, family, and household—based on “taste” (Dolado et al. 2012). Second, “network effects” may pull women into research fields in which members of their own gender are already heavily represented (Reskin 1993). Thus, women tend to be hired and promoted in fields in which gender segregation is already pronounced (Cohen et al. 1998).

In this study, we are unable to tease out selection and network effects. Our goal is more modest: namely, to give a descriptive account of the prevalence of female authorship in *Demography*. We also explore the question of whether women’s chances of being the lead or the sole author of a publication have been rising in tandem with the general increase in women’s publication activities.

## Data

To study the gender patterns of publication in research subfields in the journal *Demography*, we had to identify the gender of all of the authors and the research subfield in which each article was published (for a detailed description of the data collection and categorization procedure, see Nieberg et al. 2014). The citation information for all of the publications in *Demography* was drawn from the online JSTOR database and the Web of Science database, which covers the Science Citation Index Expanded, the Social Sciences Citation Index, and the Arts and Humanities Citation Index.

The key independent variable in our investigation is the gender of the author. However, the citation information obtained from Web of Science and JSTOR does not note the author’s gender. Using the first names provided in the literature databases, we used the program “gender.c” to identify the authors’ gender.^2^ This program contains a list of first names for all European countries, for the United States, and for some Asian countries (e.g., China, India, and Japan). According to the program’s developer, the decision rules for assigning the typical gender to each name are based on assessments provided in interviews with several native speakers and experts. The program assigns a gender to each first name if it is typically given to either males or females, and it flags unisex names. If a name was coded as unisex or as not classifiable, we identified the gender of the author manually through online research. We were unable to retrieve the gender of the author in 185 cases (4 % of the full sample). These cases were omitted from our analysis. The total sample contains 2,252 articles with 4,197 authors.

Categorizing articles by research area was the most challenging task in our analysis. After experimenting with several options, we decided to assign each article to a single category only (for a similar strategy, see Abramo et al. 2009; Dehdarirad et al. 2015; Dolado et al. 2012; Maliniak et al. 2013; West et al. 2013). Furthermore, we based the categorization of the papers on the dependent variable (see also Teachman, et al. 1993). Because demography is an applied field of research, the classification of the papers by the dependent variable seemed straightforward and easily reproducible. However, ambiguous cases remained, especially if a paper was more theoretical or covered a broad range of topics. An alternative approach might have been to use a more objective, computer-assisted classification procedure that used keywords searches. However, using a computer-based approach has disadvantages, particularly if it is based solely on a frequency count of words. For example, even if a word search showed that the term “fertility” appeared more frequently than the term “migration” in a paper, it would still be difficult to judge whether the paper was on migration or on fertility. We thus determined that a more qualitative approach that could take into consideration the chief objective of a paper (by focusing on the dependent variable) was the better option.

Before we could assign articles to subcategories, we had to create these categories. In this case as well, computer-based approaches might have been applied to generate subfields (see, e.g., Merchant 2015). We rejected this option because the core subfields in demography are well defined. Demographers generally agree that the main pillars of demographic research are fertility, mortality, migration, and methods. We therefore used these four narrow subfields as the basis for our classification procedure. After analyzing a sample of articles published in *Demography*, we extended this classification to include the following categories:

- Fertility
- Family and household
- Mortality and health^3^
- Migration
- Data and methods
- Other

To help us assign each article to one category only, we developed a list of keywords for each subfield (see Table 2 in the appendix). In a pre-test we grouped the different articles based on the list of keywords alone. However, we later determined that the inclusion of additional decision criteria would improve the classification procedure (see Nieberg et al. 2014). Among the advantages of focusing on publications in *Demography* are that the papers tend to have a similar structure, and that most are quantitative studies with a clearly defined outcome variable. For example, an article on the effect of migration on first births would have been assigned to the subfield “fertility,” while a study on the effect of fertility on migration decisions would have been assigned to the subfield “migration.” Meanwhile, a paper that explored more than one outcome—for example, fertility and mortality processes—would have been assigned to the “other” category. Some papers focused not on a cause-effect relationship between variables, but on methodological aspects, such as improved measurement or data issues. Regardless of their content, we assigned these studies to the “methods and data” category.

This classification system is clearly subjective, and assignments may vary depending on the individual rater. However, to assess the reliability of the classification procedure, we instructed three independent raters to categorize a sample of the papers based on our classification rules.^4^ The three raters independently categorized the same sample of around 200 abstracts. Based on the categorization of this sample, we calculated the coefficient kappa, which measures the degree of agreement of different raters (Cohen 1960). The referring value of kappa was found to be above 0.80, which indicates an acceptable level of agreement on the classification of the publications among these three raters.

## Methods

We first describe the development of female authorship and the publications by research subfield over time. In a second step, we estimate a multinomial logit model in which the dependent variable is the demographic subfield in which an author is publishing. To adjust for multiple observations of articles, we use robust standard errors. The main independent variables are the gender of the author and the period of publication. Because our sample is small we decided to categorize the articles by decade, although the earliest group also includes the adjacent years (1964–1979, 1980–1989, 1990–1999, 2000–2009, 2010–2014). In addition, we control for the gender of the editor over time, based on the assumption that the gender of the editor who is involved in the review process might be related to the acceptance rates of papers in more female-dominated subfields (see Table 3 in the appendix for information on the gender of the editor over time, and Table 4 in the appendix for the descriptive statistics of our sample). To investigate how the role of gender in demographic subfields evolved over time, we include an interaction of calendar year and gender in the model.

## Descriptive Results

**Fig. 1:**
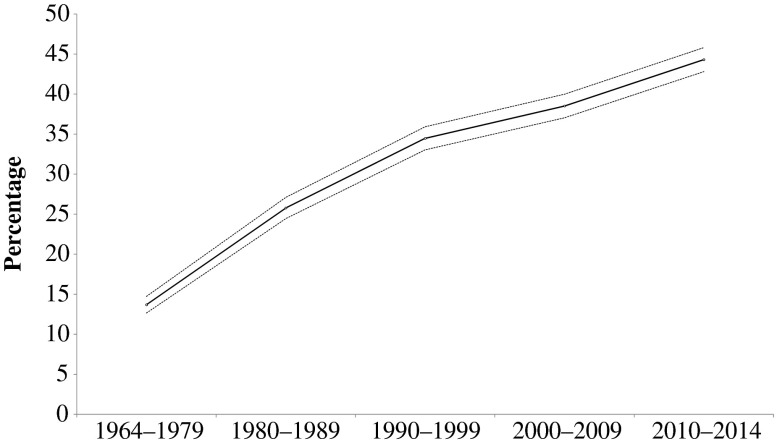
Share of female authors in *Demography* over time and 95 % confidence level. Unit of analysis is the authorship rather than the individual publication

Figure 1 displays the development over time of the share of the authors of publications in *Demography* who were women. From the first (1964–1979) to the most recent period (2010–2014), this share rose from 14 % to 44 %. These results are in line with the findings of West et al. (2013), who reported that demography is now among the most gender-equal fields of research with respect to publication output. Separate analyses of the shares of female authors among all authors who presented their research at the PAA annual meetings from 2002 to 2014 lend support to the argument that demography has become a gender-equal field of research, at least in terms of conference presentations.^5^ Unfortunately, we have no access to information on the gender breakdown of the scientists who submitted manuscripts to *Demography*. We do, however, know that the share of the students earning doctoral degrees in demography at U.S. institutions who are women has been increasing in recent decades and has been above 50 % since 2000 (National Science Foundation 2015). Female authorship in *Demography* lags behind this trend. Because we do not know how many female and male PhD recipients go on to pursue an academic career and how many authors graduated from programs in other fields, these numbers are only suggestive and do not allow us to draw firm conclusions.

**Fig. 2:**
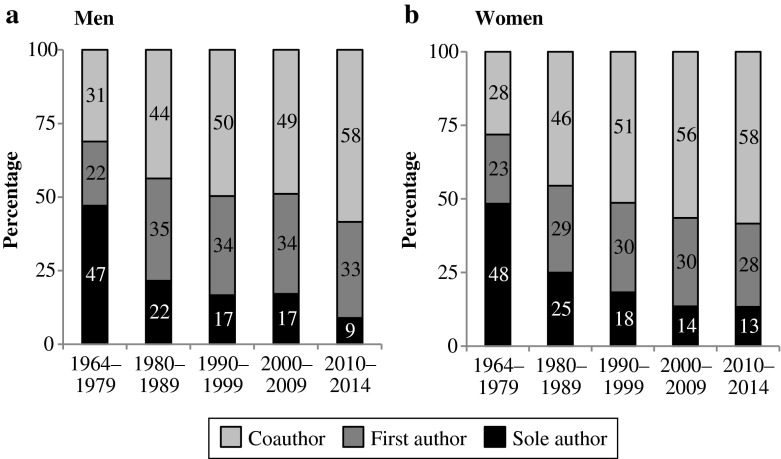
Authorship status by gender in *Demography*. Unit of analysis is the authorship rather than the individual publication

Figure 2 displays authorship status by calendar year and gender. We distinguish between sole authorship, first authorship, and coauthorship. The figure shows that publication practices have changed considerably over the years. Sole authorship was very common in *Demography* in 1964–1979: during that period, about half of all authors were sole authors. Since then, the prevalence of sole authorship has declined rapidly. In the period 2010–2014, for example, only 13 % of all female authors were sole authors. At the same time, the share of those being a first author in a multiple-authored paper rose slightly. Interestingly, women and men were equally likely to have published a paper as the sole or first author.^6^

**Fig. 3:**
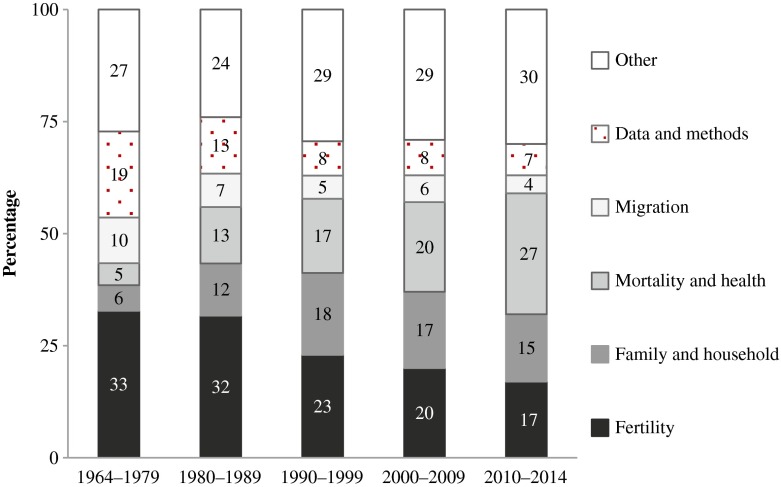
Evolution of subfields of publication in *Demography* over time. Unit of analysis here is the individual publication

When considering the results of the analyses of research subfields over time, it is important to keep in mind that our focus is on the outcome variable. Taking this narrow view, we see some striking trends in Fig. 3, which maps the evolution of subfields. First, the relative significance of papers assigned to the “data and methods” category has declined over time: as a percentage of all of the papers published, the share of the papers that were in the subfield of “data and methods” fell from 19 % in 1964–1979 to 7 % in 2010–2014. However, we cannot rule out the possibility that the decline in the number of papers in this category is an artifact attributable to the increased tendency to integrate methodological considerations into papers that also have a substantive topic. Looking at the number of articles that used an outcome variable related to one of the traditional demographic subfields of “fertility,” “mortality and health,” and “migration” reveals that the number of publications in the subfield of “mortality and health” has grown especially rapidly in recent decades. By contrast, despite the obvious social policy relevance of migration, the number of publications on this topic has remained small (see also Kirk 1968; Rößger 2014). The dearth of publications on migration may be attributable to a lack of data available to researchers in this subfield. However, we assigned some of the articles that dealt with the social aspects of migration—such as discrimination against migrants or the integration of them—to the “other” category in our investigation. This reveals the main shortcoming of our classification procedure, which assumes that each paper included a well-defined outcome variable. It is interesting to observe that articles with a fertility-related outcome variable have become less prevalent in *Demography*. Whereas the share of papers with a focus on “fertility” has declined, the share of papers analyzing an outcome variable in the area of “family and household” research has expanded in recent years, accounting for 15 % of all publications in *Demography* in 2010–2014.

## Multivariate Results

Table 1 displays the results of the multinomial logistic regression model with the subfield of study as the dependent variable. We chose “other” as the base category, given that descriptive statistics show that the gender composition in this subfield is almost constant over time. Our period indicator shows a pattern that is in line with the previous descriptive statistics. Over time, the relevance of articles in the categories “data and methods,” “migration,” and “fertility” declined; the relevance of papers in the categories “mortality and health” and “family and household” increased.

**Table 1:** Results from multinomial logistic regression with “subfield of publication” as a dependent variable, base outcome: Other field. Relative risk ratios (RRR) and *z* statistics

	Data and Methods		Mortality and Health		Migration		Fertility		Family and Household	
	RRR	*z* Statistics	RRR	*z* Statistics	RRR	*z* Statistics	RRR	*z* Statistics	RRR	*z* Statistics
Male (ref.)										
Female	0.74	–2.24*	0.78	–2.14*	0.66	–2.42*	1.02	0.16	1.61	4.15**
1964–1979 (ref.)										
1980–1989	0.75	–1.24	3.29	4.31**	0.78	–0.92	1.06	0.33	1.90	2.39*
1990–1999	0.42	–3.40**	3.39	4.65**	0.45	–2.46*	0.63	–2.46*	2.33	3.36**
2000–2010	0.46	–2.23*	3.10	3.57**	0.44	–2.72**	0.48	–2.92**	2.68	3.23**
2010–2014	0.52	–2.30*	6.41	7.10**	0.36	–2.77**	0.44	–3.68**	1.79	2.14*
Editor Male (ref.)										
Editor Female	1.36	1.23	0.83	–0.78	1.67	2.02*	1.27	1.28	1.61	2.09*
Editor Mixed	1.44	0.90	1.83	2.03*	2.29	2.36*	1.36	1.09	0.99	--0.02
Constant	0.69	–2.92**	0.18	–8.71**	0.39	–6.11**	1.32	2.59**	0.20	--8.22**

*Note:* Robust standard errors account for the clustering of authors in single publications. Editor mixed: multiple editors, at least one male and one female editor.

*p < .05; **p < .01

**Fig. 4:**
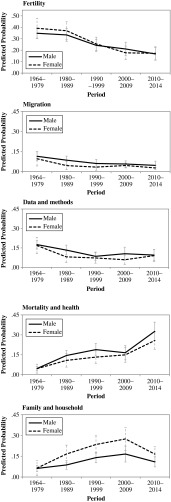
Results of multinomial logit model. Predicted probabilities of publication for men and women by period and subfield and 95 % confidence bounds. Robust standard errors are included to account for the clustering of authors in single publications. Detailed regression results will be provided upon request

Our key variable of interest is gender. We find that women were less likely than men to have published papers in the categories “data and methods,” “migration,” and “mortality and health.” We find no difference by gender in publications on fertility research. Women were substantially more likely than men to have published papers with outcome variables in “family and household” (the relative risk ratio was 61 % higher for women than for men). To help determine whether this pattern has changed over time, Fig. 4 in the appendix displays the results from an interaction of gender and period.^7^ The figure shows a significant overrepresentation of women authors in the category “family and household” beginning in the 1980s. Since then, women have had higher relative risk ratios of publishing studies with a family- or household-related outcome variable.

The model also controls for the gender composition of the editors. We expected to find that female editors would be more supportive of publications in categories in which there are more female authors. However, we do not find strong evidence to support this assumption. Although female editorship was associated with an increase in the chances that a paper would be published in the category “family and household,” the coefficient was significant at only the 5 % level. In addition, female or mixed editorship increased the chances that a paper with a migration-related outcome variable would be published. However, the authorship of papers in this category was not dominated by women.

## Discussion

In this study, we investigated the development of demographic research subfields and gendered publication practices by examining publications in *Demography* spanning the 50-year period of 1964–2014. More than 40 % of all of the authors who published in *Demography* in the period 2010–2014 were female. This result corroborates previous findings that demography is more gender-balanced than most other fields of research. Moreover, we did not find that women have been disadvantaged in terms of coauthorship practices. Over the period studied, women were as likely as men to have been the sole author of a paper or the first author of a paper with multiple authors. Based on these simple descriptive statistics, it is tempting to conclude that *Demography* is a gender-equal journal.

However, some caution is warranted. Our findings indicate that although women are authoring more papers than in the past, they have not yet reached parity with men. The trend toward female authorship in demographic research has so far been positive, but we should carefully observe how this trend develops in the future. Other studies have, for example, shown that in several fields, the share of PhD recipients who are women has plateaued at levels below 50 % (England 2010:160). A closer inspection of the subfields of publications in *Demography* also reveals gender differences by outcome variables. Our multivariate analyses indicate that women authors have been overrepresented in the “family and household” category, but they have been less likely to publish in the “mortality and health,” “migration,” and “data and methods” categories. Our scheme for categorizing research subfields is limited in a number of ways, including by the narrow identification based on the outcome variable and the large fraction of unclassifiable papers. We believe, however, that our findings provide evidence of some segregation by gender in the publications in *Demography*.

These results raise several questions. For example, are our findings representative of demography as a field of research, and do they reflect publication patterns in other journals, such as *Population and Development Review* and *Population Studies*? Furthermore, are the gendered publication patterns in *Demography* an indication of the exclusion of women (and men) from specific research subfields, or do they instead reflect different research interests among female researchers? We have shown in this article that gendered publication practices exist, but we have been unable to identify the mechanisms that produced these patterns. Our data also do not allow us to address the issue of whether gendered publication practices affect the academic advancement of women in demographic research. Moreover, examining the number of female submissions might have enabled us to assess whether female authors were subject to discrimination in the review process, but this information was not available. Although we were unable to answer these substantive questions, we hope that this descriptive paper motivates others to conduct more fine-grained analyses of gendered publication activities in demographic research.

## Appendix

**Table 2:** List of keywords for classification scheme

Subfield	Keyword
Fertility	Births, birth rate, childbearing, childbearing desires/preferences, pregnancy spacing of births, birth interval, reproductive health, sexual health/HIV, family planning (programs), contraception, sterility, abortion, infanticide, sex ratio, fecundity, miscarriage, fetal loss, abortion, breast-feeding, postpartum, amenorrhea
Family and Household	Family, partnerships, marriage, household, leaving home, institutional child care/parental leave, elderly care, (gendered) division of labor, child support, child well-being, intermarriage, kinship, homogamy
Mortality and Health	Mortality, survival, causes of death, infant/neonatal mortality, life expectancy, health, aging, disability, transition to disability/care, care need, healthy life expectancy, healthy behavior/risk behavior, birth weight, BMI/obesity, height
Migration	Migration processes, residential mobility, internal/international migration
Data and Methods	Methods, statistics, calculus, dispersion, Bayesian forecasting, modeling/models, stable population/stationary population, measures/measurement, population composition, population projections, population growth, reporting errors, survey methodology, data collection procedures
Other	Education, language/cognitive abilities, developmental outcomes, labor market, transition to retirement, pension policy, wealth/poverty, income, income mobility, neighborhood, residential distribution, ethnic inequality, integration/segregation, socioeconomic status, environment, urbanization, domestic violence, social networks, crime, demography as a field of research, review articles, replies/comments, unclassifiable

**Table 3:** Gender of the editor of Demography

Year	Gender
1964–1965	M
1966	M
1967–1968	M
1969–1971	F
1972	M
1973–1975	M
1976–1978	M
1979–1980	M
1981–1984	M
1985–1987	F
1988–1990	M
1991–1993	M
1994–1995	F
1996–1998	M
1999–2001	M/F
2002–2004	M
2005–2007	M/F
2008–2010	M
2011–2013	M
2014–2016	F

*Note:* M = male editor; F = female editor; M/F = multiple editors, with at least one male and one female.

**Table 4:** Descriptive statistics

	Authorships		Publications	
	*n*	Proportion	*n*	Proportion
Male	2,917	.70	1,598	.71
Female	1,280	.30	654	.29
1964–1979	1,008	.24	720	.32
1980–1989	772	.18	424	.19
1990–1999	818	.20	403	.18
2000–2009	818	.20	380	.17
2010–2014	781	.19	325	.14
Editor Male	2,967	.71	1,646	.73
Editor Female	691	.16	361	.16
Editor Mixed	539	.13	245	.11
Fertility	1,122	.27	590	.26
Family and Household	553	.13	280	.12
Mortality and Health	656	.16	316	.14
Migration	285	.07	161	.07
Data and Methods	485	.11	281	.13
Other	1,096	.26	624	.28
Number of Authorships	4,197	1.00	––	––
Number of Publications	––	––	2,252	1.00

*Note:* Editor mixed = multiple editors, with at least one male and one female editor.

**Table 5:** Results from multinomial logistic regression with “subfield of publication” as a dependent variable: Interaction effect of gender and period. Relative risk ratios (RRR) and *z* statistics

	Data and Methods		Mortality and Health		Migration		Fertility		Family and Household	
	RRR	*z* Statistics	RRR	*z* Statistics	RRR	*z* Statistics	RRR	*z* Statistics	RRR	*z* Statistics
Male, 1964–1979 (ref.)										
Male, 1980–1989	0.81	–0.88	3.45	4.29**	0.84	–0.63	1.06	0.30	1.69	1.79^†^
Male, 1990–1999	0.41	–3.24**	3.59	4.62**	0.49	–2.10*	0.63	–2.22*	2.12	2.77**
Male, 2000–2009	0.53	–1.80^†^	3.06	3.33**	0.43	–2.76**	0.52	–2.37*	2.47	2.71**
Male, 2010–2014	0.51	–2.16*	6.95	6.90**	0.41	–2.19*	0.47	–2.95**	1.82	2.02*
Female, 1964–1979	0.98	–0.06	1.02	0.04	0.88	–0.36	1.19	0.74	1.15	0.28
Female, 1980–1989	0.49	–1.98*	2.51	2.50*	0.45	–1.97*	1.18	0.67	3.14	3.43**
Female, 1990–1999	0.35	–3.04**	2.57	2.93**	0.27	–3.05**	0.67	–1.75^†^	3.65	4.43**
Female, 2000–2009	0.28	–3.16**	2.73	2.89**	0.34	–2.56*	0.44	–2.91**	4.09	4.29**
Female, 2010–2014	0.43	–2.46*	4.86	5.37**	0.21	–3.37**	0.44	–3.09**	2.50	3.02**
Editor Male (ref.)										
Editor Female	1.36	1.23	0.83	–0.76	1.68^†^	2.05*	1.27	1.29	1.61	2.08*
Editor Mixed	1.43	0.90	1.84	2.04*	2.31	2.39*	1.36	1.08	0.99	–0.02
Constant	0.66	–3.10**	0.18	–8.55**	0.37	–6.13**	1.29	2.30*	0.22	–7.57**
*n* (authors)	4,197									
*n* (articles)	2,252									
Log-Likelihood Null Model	–7,103.29									
Log-Likelihood Full Model	–6,834.03									

*Note:* Robust standard errors account for the clustering of authors in single publications. Editor mixed = multiple editors, with at least one male and one female editor.

^†^ *p* < .10; **p* < .05; ***p* < .01

## Footnotes

1 The authors used all of the publications archived in JSTOR.

2 The copyright (2007–2008) for this program lies with Jörg Michael, Adalbert-Stifter-Str. 11, 30655 Hannover, Germany. The program is subject to the GNU Lesser General Public License (LGPL), as published by the Free Software Foundation.

3 Initially, “mortality and health” was broken down into two separate categories. We subsequently chose to combine the two categories because of sample size issues.

4 In order to classify the papers, the raters used the titles and the abstract. The authors’ gender was not known to the raters.

5 It would be interesting to compare this development with the total share of demographers who are women or with the percentage of members of the PAA who are female. Unfortunately, we do not have reliable time series information of this kind.

6 We also did not find any evidence that male authors were more likely than female authors to have been the last author of a paper with multiple authors (results not shown here).

7 The predicted probabilities for the interaction effect can be found in Table 5 in the appendix.
